# Ultrasound-assisted extraction enhances recovery of antioxidant-rich carbohydrate fraction from mixed microalgae species

**DOI:** 10.1016/j.ultsonch.2025.107656

**Published:** 2025-10-30

**Authors:** Eldwin Ze Hao Ooi, Eng-Seng Chan, Cher Pin Song, Janarthanan Pushpamalar, Yee-Ying Lee

**Affiliations:** a School of Science, Monash University Malaysia, Jalan Lagoon Selatan, Bandar Sunway, 47500 Subang Jaya, Selangor, Malaysia; b Department of Chemical Engineering, School of Engineering, Monash University Malaysia, Jalan Lagoon Selatan, Bandar Sunway, 47500 Subang Jaya, Selangor, Malaysia; c MIPO Biorefinery Laboratory, Monash University Malaysia, Jalan Lagoon Selatan, Bandar Sunway, 47500 Subang Jaya, Selangor, Malaysia

**Keywords:** Ultrasound, Microalgae, Antioxidant, Carbohydrate recovery, Physicochemical properties

## Abstract

The effects of ultrasonic processing parameters on the functional and physicochemical properties of microalgae carbohydrates remain poorly understood, despite evidence from plant-based extractions showing that sonication conditions strongly influence bioactive compound yield and antioxidant activity from carbohydrates. This study investigated the effects of ultrasonic-assisted extraction (UAE) parameters on the yield, composition, and antioxidant activity of carbohydrates extracted from defatted mixed microalgae biomass. A single-factor approach was employed to test different extraction times (10–30 min), amplitudes (20–100 %), and solid-to-solvent ratios (1:10–1:30 g/mL). All analyses were conducted in triplicate, and statistical significance was determined at p < 0.05. Results showed that processing time was the most influential factor in improving antioxidant activity. Under optimal UAE conditions (25 min, 100 % amplitude, 1:15 g/mL), the extract contained significantly higher uronic acid, sulphate, and fibre contents (p < 0.05) compared to hot water extraction. These changes corresponded to stronger antioxidant activity (∼35 % increase in DPPH radical scavenging). Correlation analysis further revealed that uronic acid had the strongest positive influence on antioxidant activity. In conclusion, UAE improved both the yield and antioxidant properties of microalgae carbohydrates compared with hot water extraction. With its high fibre content and bioactivity, the extract may have potential applications as a functional food ingredient.

## Graphical abstract

**Figure:**
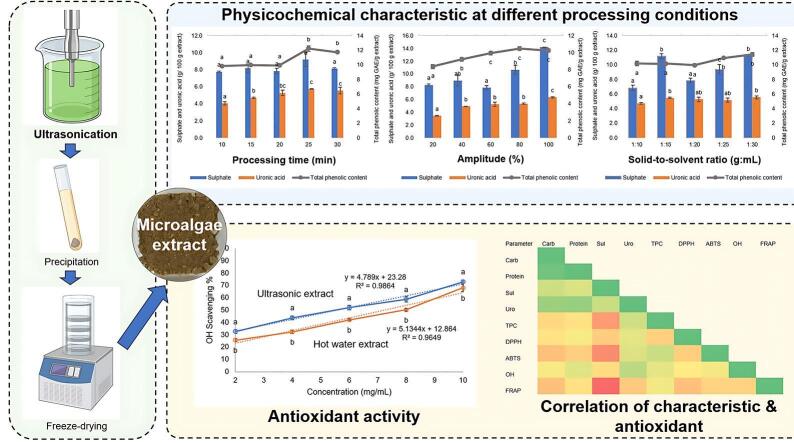

## 1 Introduction

Microalgae and cyanobacteria are photosynthetic microorganisms that are eukaryotic and prokaryotic, respectively. The most widely commercialised species include *Chlorella vulgaris*, *Arthrospira platensis* (Spirulina) and *Nostoc commune* (cyanobacteria) [1]. Microalgae are efficient CO_2_-fixing microorganisms with rapid growth and the capacity to synthesise diverse bioactive molecules (lipids, proteins, carbohydrates, vitamins and phytonutrients) [2].

Among these bioactive molecules, microalgae carbohydrates have been shown to possess multiple functionalities, including antioxidant, anti-cancer, and antibacterial activities [[3], [4], [5]]. The beneficial properties of polysaccharides can be attributed to their monosaccharide composition, molecular weight, sulphate and uronic acid content and type of glycosidic bonds [6]. For example, sulphate groups with their complex structural characteristics enable them to interact with biomolecules, preventing inflammation and promoting wound healing [7]. Furthermore, microalgal carbohydrates are predominantly non-digestible carbohydrates. Their high viscosity can delay gastric emptying and prolong satiety, thereby contributing to their role as natural anti-obesity agents [8]. Furthermore, their resistance to digestion enables fermentation by colonic microbiota, exerting prebiotic effects through the selective stimulation of beneficial microbial populations [9]. Collectively, these attributes underscore the potential of microalgal polysaccharides as a multifunctional food ingredient.

To extract the carbohydrates, the extraction technology is important to ensure that the rigid microalgae cell wall and its structural components are lysed to ensure effective extraction and yield. Conventionally, hot water and acid/alkali extraction are the most common extraction methods; however, they require long extraction times and the usage of harsh chemicals, which are not environmentally friendly and severely impact economic costs at the industrial scale. Hence, green technologies such as ultrasonication, microwave, high-pressure homogenisation and pulsed electric field have been emerging as the method of choice for microalgae carbohydrate extraction [9]. These technologies remove the need for harsh chemicals, require lower energy consumption due to their shorter processing times and produce less waste.

Ultrasonic-assisted extraction (UAE) is a mechanical extraction method that employs ultrasound waves with frequencies ranging from 20 kHz to 10 MHz to induce cavitation for extraction [10]. Ultrasonication induces acoustic cavitation, which generates microbubbles in the solvent. These bubbles grow with succeeding cycles of cavitation and eventually collapse, producing high temperatures, pressures and shock waves that damage or rupture the microalgae cell wall, facilitating the entry of solvents into cells and increasing mass transfer rate and extraction efficiency [10]. UAE has advantages compared to other green approaches in that it can also achieve significant yields even at lower temperatures, with lower volumes of extraction solvent, making it suitable for the extraction of heat-sensitive compounds.

Various studies have been conducted to extract carbohydrates from non-defatted microalgae species such as *Spirulina platensis*, *Phaeodactylum tricornutum*, and *Chlorella vulgaris* via ultrasonication. A carbohydrate yield of 8–17 % (g/100 g biomass) has been obtained, which is comparable to microwave and acid/alkali extraction methods [3,6]. Additionally, UAE has been found to enhance the functional properties (e.g. antioxidant and anti-diabetic) of the extracted polysaccharides [5,11]. To the best of our knowledge, existing literature largely compares different extraction methods for carbohydrate yield, primarily in the context of bioethanol production [12]. Whereas the impact of ultrasonic processing variables on the functional properties of microalgae carbohydrates, such as antioxidant activity and its relationship to physicochemical properties (sulphate, uronic acid), remains inadequately understood. Past studies on plant leaves extraction have found that ultrasonication time and amplitude significantly enhanced polyphenol yields and antioxidant activity. Extraction of *Melastoma malabathricum* leaves at 32 °C, 16  min and 70 % ethanol yielded 96 % DPPH scavenging, while beyond 50 % amplitude and 5 min lowered polyphenol yields in *Ocimum basilicum* leaves due to higher temperatures causing thermal degradation [13,14]. Understanding the effects of these parameters is crucial for maximising the concentration of bioactive compounds while minimising solvent consumption, energy input, and processing time, thereby meeting sustainability and economic targets that drive modern green‑technology adoption. Hence, this study aims to study the effect of various ultrasonic processing conditions (solid-to-solvent ratio, processing time, ultrasonic amplitude) on the yield as well as physicochemical and antioxidative properties of the microalgae carbohydrate extract. The properties that contribute to the antioxidant activity of the extract were also investigated. The research work is significant as it highlights the potential of the carbohydrate extract as a nutrient-dense functional component in human nutrition, such as nutraceuticals and dietary supplements [1].

## 2 Material and methods

### 2.1 Materials

Defatted, dried mixed microalgae biomass (*Chlamydomonas reinhardtii*, *Chlorella* sp. and *Nostoc commune*, moisture content = 9.357 ± 0.136 %) was supplied by Petronas Research Sdn. Bhd. (Selangor, Malaysia). All extractions were done using the same sample batch. All chemical reagents and solvents used were of analytical grade and purchased from Sigma-Aldrich (Burlington, United States).

### 2.2 Extraction of microalgae carbohydrates

#### 2.2.1 Ultrasonic-assisted extraction

Ultrasonic-assisted extraction was performed using a CPX500 Ultrasonic Processor (500 W, 20 kHz frequency), attached with an ultrasonic probe VCX750 with a diameter of 1 cm (Cole-Parmer, Illinois, United States). 5 g microalgae powder was added to water at different solid-to-water ratios (1:10, 1:15, 1:20, 1:25, 1:30 g/mL, w/v), hydrated and mixed thoroughly for 5 mins. Then the probe was immersed in the solvent to a depth of 1 cm, and ultrasonication was conducted at various processing times (10, 15, 20, 25, 30 min) and ultrasonic amplitudes (20, 40, 60, 80, 100 %). When manipulating one variable, the other variables were fixed at 1:20 g/mL, 20 min or 60 % amplitude. The extraction was maintained at 25–30 °C using an ice bath and monitored using a temperature probe. After extraction, the mixture was centrifuged at 8801 rcf for 15 min, and the supernatant was decanted for precipitation. Using the conditions with the highest yield, identified through the single-factor study, the extract prepared using ultrasonic-assisted extraction (UAE) was compared with an extract prepared using conventional hot water extraction (HWE).

#### 2.2.2 Conventional hot water extraction (HWE)

HWE was done using a microalgae solid-to-water ratio of 1:25 g/mL at 80 °C for 2 h with constant stirring using a temperature-controlled hotplate set at 80 °C (IKA, Selangor, Malaysia). After extraction, the mixture was centrifuged at 8801 rcf for 15 min at room temperature, and the supernatant was decanted for precipitation.

#### 2.2.3 Ethanol precipitation

The water-soluble fraction was isolated from the supernatant by adding 95 % ethanol at a 1:4 (v/v) supernatant: ethanol ratio, then kept at 4 °C for 24 h. Then, the mixture was centrifuged at 3434 rcf for 15 min to collect the precipitate, which was re-dissolved in water prior to freeze drying to produce the microalgae extract powder. The powder was stored at −20 °C until further analysis.

### 2.3 Extract characterisation

#### 2.3.1 Physicochemical properties

The carbohydrate yield of the extract was determined via the phenol-sulphur method following the protocol described by DuBois et al. [15]. The protein yield was measured via the Kjeldahl method according to AOAC Official Method 2001.11, using a conversion factor of 4.78 as determined by Lourenço et al. [16]. Both the carbohydrate and protein yields were calculated using formulas (1) and (2), respectively.

(1) $$Carbohydrate\;yield\;\left(g/100\;g\;biomass\right)\;=\;\frac{\mathrm{Mass}\;of\;carbohydrate\;in\;extract}{\mathrm{Mass}\;of\;microalgae\;biomass}\times 100\%$$

(2) $$Protein\;yield\;\left(g/100\;g\;biomass\right)\;=\;\frac{\mathrm{Mass}\;\mathrm{of}\;\mathrm{protein}\;\mathrm{in}\;\mathrm{extract}}{\mathrm{Mass}\;\mathrm{of}\;\mathrm{microalgae}\;\mathrm{biomass}}\times 100\%$$

The insoluble and soluble dietary fibre content of the extracts was determined using the enzymatic–gravimetric standard protocol according to AOAC Official Method 2011.25.

The sulphate content in the extract was determined using the microplate barium chloride-gelatine method as described by Torres et al. [17]. Sodium sulphate was used to plot the calibration curve (0–0.6 mg SO_4_^2-^/mL) (Fig. S1a) for quantification. The results were expressed as a percentage of mass of uronic acid to sample mass (g/ 100 g extract).

The uronic acid content of the extract was measured using 0.15 % m-hydroxydiphenyl reagent (Sigma 262250) reacting with the sample mixed with 0.0125 M sulphuric acid/tetraborate solution [18]. The absorbance at 520 nm was measured using an Infinite M200 Pro Microplate Reader (Tecan, Männedorf, Switzerland), and quantification was done using a standard curve of glucuronic acid (0–100 µg/mL) (Fig. S1b), and the results were expressed as a percentage of mass of uronic acid to sample mass (g/ 100 g extract).

To measure the total phenolic content (TPC) of the extract, 100 μL of 5 mg/mL extract in water was mixed with 100 μL of 50 % (v/v) Folin-Ciocalteu solution (Sigma 47641). Then, 3 mL of 2 % (w/v) sodium carbonate was added to the mixture, and the samples were incubated at room temperature, in the dark, for 1 h [19]. The absorbance at 750 nm was measured using a Secomam Prim Light Spectrophotometer (AHS Laboratory Supplies, Kuala Lumpur, Malaysia), and TPC was quantified using a gallic acid standard calibration curve (0–200 µg/mL) (Fig. S1c). The results were expressed as milligrams of Gallic Acid Equivalent per gram of extract (mg GAE/g extract).

#### 2.3.2 Antioxidant activity of the extract

Prior to measuring the antioxidant activity of the extracts, the dried powder was dissolved in water at a concentration of 4 mg/mL. The 2,2-diphenyl-1-picrylhydrazyl radical (DPPH) (Sigma D9132) scavenging activity was measured using a spectrophotometer with a wavelength of 517 nm according to the method by Yu et al. [6]. Whereas the hydroxyl radical (OH•) scavenging activity was determined using a spectrophotometer with a wavelength of 510 nm according to the method by Song et al. [20]. The scavenging activity for both assays was calculated using the formula below:

(3) $$\mathrm{Radical}\;scavenging\;activity\;\left(\%\right)=\left[1-\left(A_{\mathrm{sample}}-A_{\mathrm{blank}}\right)/A_{\mathrm{control}}\right]\times 100\%$$

where A_sample_ = the absorbance of the sample, A_blank_ = the absorbance of sample without the reagent, A_control_ = the absorbance of reagent and water (without sample).

The 2,2′-azino-bis(3-ethylbenzothiazoline-6-sulfonic acid) radical (ABTS) (Sigma A1888) scavenging activity was measured using a microplate reader with a wavelength of 730 nm following the method adopted from Chen et al. [11]. The scavenging capacity was calculated as follows:

(4) $$\mathrm{ABTS}\;scavenging\;capacity\;\left(\%\right)\;=\;\left(A_{0}-\;A_{1}\right)/A_{0}\times 100\%$$

where A_0_ is the blank control absorbance (without sample), A_1_ is sample absorbance.

The ferric reducing antioxidant power (FRAP) assay was conducted using a microplate reader with a wavelength of 593 nm according to the method from Chen et al. [11]. Iron (II) sulphate solution was used as the standard to obtain a calibration curve (0–0.4 μmol FeSO_4_/mL) (Fig. S1d), and the FRAP value of the samples was expressed as μmol FeSO_4_/g extract.

The UAE and HWE were tested at concentrations ranging from 0.5 to 10 mg/mL to determine the half-maximal inhibitory concentration (IC_50_), along with ascorbic acid as a reference standard. IC_50_ values were determined by plotting radical scavenging activity against extract concentration using Microsoft Excel. A linear regression model was applied to the dose–response data, and the IC_50_ was calculated from the fitted equation. The corresponding R^2^ values, indicating goodness of fit, are reported in the figures.

#### 2.3.3 FTIR analysis of extracts

Attenuated Total Reflection Fourier Transform Infrared Spectroscopy (ATR-FTIR) scans of the extracts were carried out using a PerkinElmer Spectrum Two FTIR machine (PerkinElmer, Shelton, United States) with a 4000–400 cm^−1^ spectral scanning range. The dried sample was placed on the ATR crystal, completely covering it, and the spectrometer was set to 16 scans to obtain the spectra of the sample.

#### 2.3.4 SEM of microalgae biomass after extraction

The surface morphology of the microalgae biomass powder after HWE and UAE was observed using a S-3400 N Type II scanning electron microscope (SEM) (Hitachi, Tokyo, Japan) with a constant voltage of 5  kV. Prior to viewing, the samples were sputtered with platinum using a Q150R S sputter coater (Quorum, East Sussex, United Kingdom). The micrographs were taken at × 2000 magnification.

### 2.4 Statistical analysis

All samples were analysed in triplicate or duplicates. Data are presented as mean ± standard deviation. The data’s normality was validated using the Kolmogorov–Smirnov test and was evaluated using one-way ANOVA via Statistical Package for the Social Sciences (SPSS) Statistics Version 29. Post-hoc Tukey test was used to determine statistically significant differences at a p < 0.05 level. Pearson correlation between the physicochemical properties and the antioxidant activity of the extract was also calculated to investigate the influence of different physicochemical properties on the functional properties of the microalgae extract.

## 3 Results and discussion

### 3.1 Effect of ultrasonic processing conditions on extract yield and chemical properties

Ultrasonic extraction is an effective extraction method that uses high-frequency sonication waves to break the cellular structure. Higher cell fragmentation promotes the release of structural carbohydrates, allowing more cellular contents, such as proteins and non-structural carbohydrates, to be extracted into the solvent [21]. Primarily, these compounds exhibit antioxidative properties [4]. Hence, the effects of ultrasonic processing conditions (ultrasonic processing time, ultrasonic amplitude, microalgae solid-to-solvent ratio) on carbohydrate yield and chemical properties of the extract were studied.

#### 3.1.1 Processing time

**Fig. 1:**
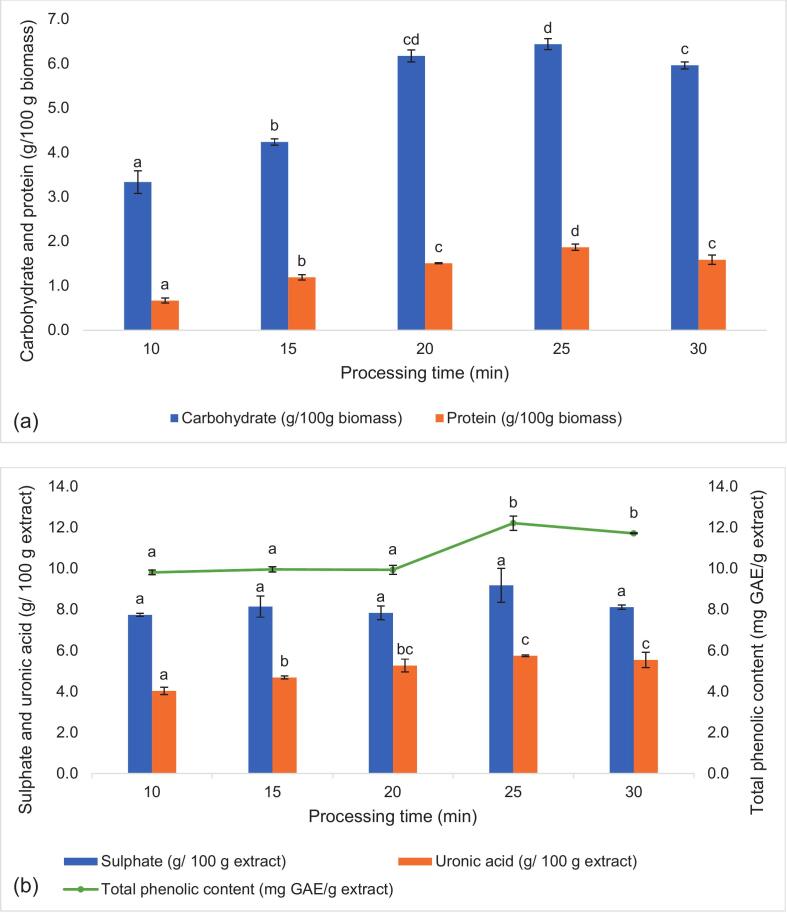
Carbohydrate and protein yield (a), along with sulphate, uronic acid and total phenolic content (b) of the microalgae extracts produced under varying processing time (10–30 mins). Data is presented as mean ± SD of three replicates. Values with different letters (^a,b,c,d^) within the same analysis are significantly (p < 0.05) different.

The carbohydrate and protein yield increased gradually from 10 to 25 min, where they reached a maximum yield of 6.438 and 1.868 g/100 g biomass, respectively, before decreasing by 7.41 % and 15.14 % for carbohydrate and protein, respectively (Fig. 1a). A similar observation was reported by Khawli et al. [19], where carbohydrate yield decreased from 2.13 % to 1.90 % when the extraction time was extended from 15 to 30 min. An increase in processing time led to a higher cell disruption rate, which allowed the release of more compounds into the solvent [11,21]. However, beyond that, no improvement in yield was observed, as maximum cell disruption has been achieved. Similar trends were observed for the uronic acid of the extract (Fig. 1b). Meanwhile, the sulphate content increased with extraction time, reaching a maximum of 9.185 g/100 g extract at 25 min. With a longer processing time, the gradual dissolution and diffusion of functional carbohydrates increased, leading to enhanced uronic acid and sulphate yield [6]. It was also noted that the extract contains trace amounts of TPC (9.813 to 12.218 mg GAE/g extract). These phenolic compounds, including flavonoids and phenolic acids, are secondary metabolites produced by microalgae in response to stress, acting as potent antioxidants that help the microorganism to survive and adapt to its surroundings [22]. It was found that after 30 min of extraction, TPC decreased (Fig. 1b), which could be attributed to prolonged ultrasonic cavitation generating free radicals that caused the degradation of TPC. It was previously reported that as ultrasonication progressed, the radical concentration increased, and after a certain threshold, degradation of phenolic compounds became significant [10,23]. This aligns with past studies of UAE on polyphenols, where at 80 % amplitude, the radical indicator glutathione decreased by 29.2 % after 30 mins of sonication [24]. Aloisio et al. [14] also reported that beyond 20 min of sonication, harsh sonochemical effects caused a significant drop in TPC when extracting *Ocimum basilicum* leaves. Hence, 25 min was considered sufficient to preserve the functional properties of the carbohydrate.

#### 3.1.2 Ultrasonic amplitude

**Fig. 2:**
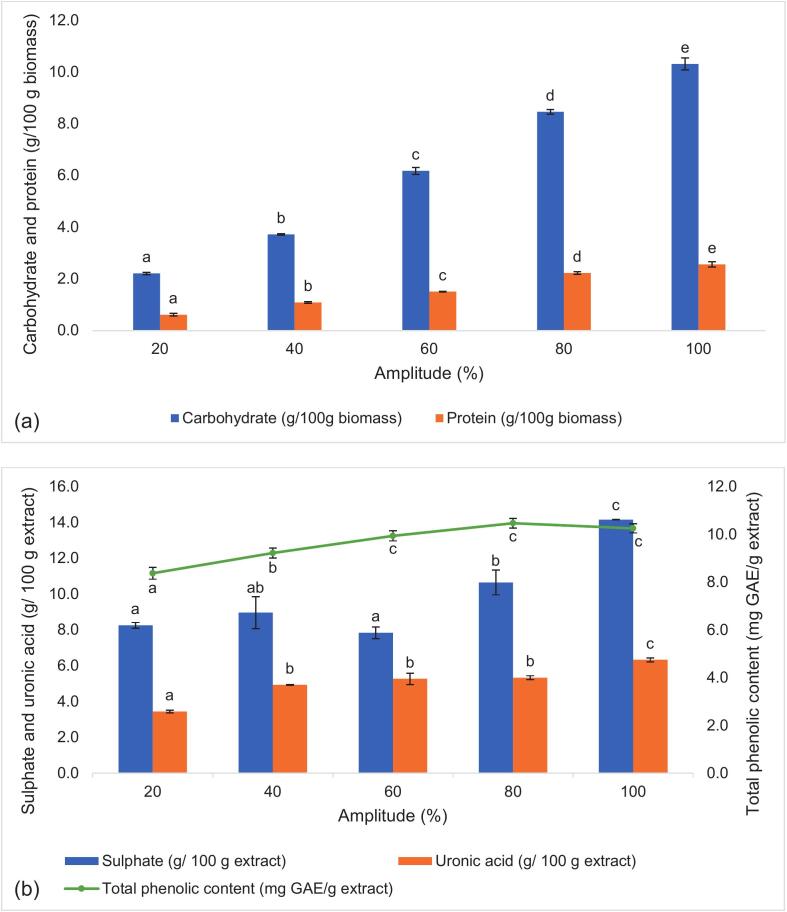
Carbohydrate and protein yield (a), along with sulphate, uronic acid and total phenolic content (b) of the microalgae extracts produced under varying ultrasonic amplitude (20–100 %). Data is presented as mean ± SD of three replicates. Values with different letters (^a,b,c,d^) within the same analysis are significantly (p < 0.05) different.

The increase in ultrasonic amplitude led to a significant increase in carbohydrate and protein yield (Fig. 2a), reaching a maximum yield of 10.31 g carbohydrate/100 g biomass at 100 % amplitude. Similarly, sulphate and uronic acid content significantly increased (p < 0.05) as the ultrasonic amplitude increased, reaching a maximum of 14.159 and 6.328 g/100 g extract, respectively (Fig. 2b). Amplitude is defined as the maximum distance travelled by the ultrasonic horn. By increasing the amplitude, more energy was introduced to the system, creating a larger cavitation size that caused a more violent bubble collapse [10]. This ultimately increased the rupture rate of the microalgae cells, allowing more intracellular compounds to be released. Maximum amplitude proved effective in extracting functional groups attached to the microalgae cell wall, such as sulphate and uronic acids, as the increase in amplitude led to more cell wall disruption, which enhanced the extraction efficiency [25]. The obtained results were consistent with Zhao et al. [26] and Lupatini et al. [21], who reported a positive relationship between yield and amplitude. Other studies showed that excessive ultrasonic power introduced to the system led to a lower polyphenol yield, which was also observed in this study, where TPC decreased at 100 % amplitude [11,27]. This occurred because higher cavitation promoted free radical formation, which can accelerate the oxidation of phenolic compounds. However, a short processing time (20 min) was used in this study, which may have prevented excessive free radicals that could damage the biomolecules [27]. Based on the results, it can be inferred that the polysaccharide components (carbohydrates, uronic acids, and sulphates) remained stable or increased under higher amplitudes, suggesting that ultrasonic-induced degradation was more pronounced in phenolic compounds than in polysaccharides.

#### 3.1.3 Solid-to-solvent ratio

**Fig. 3:**
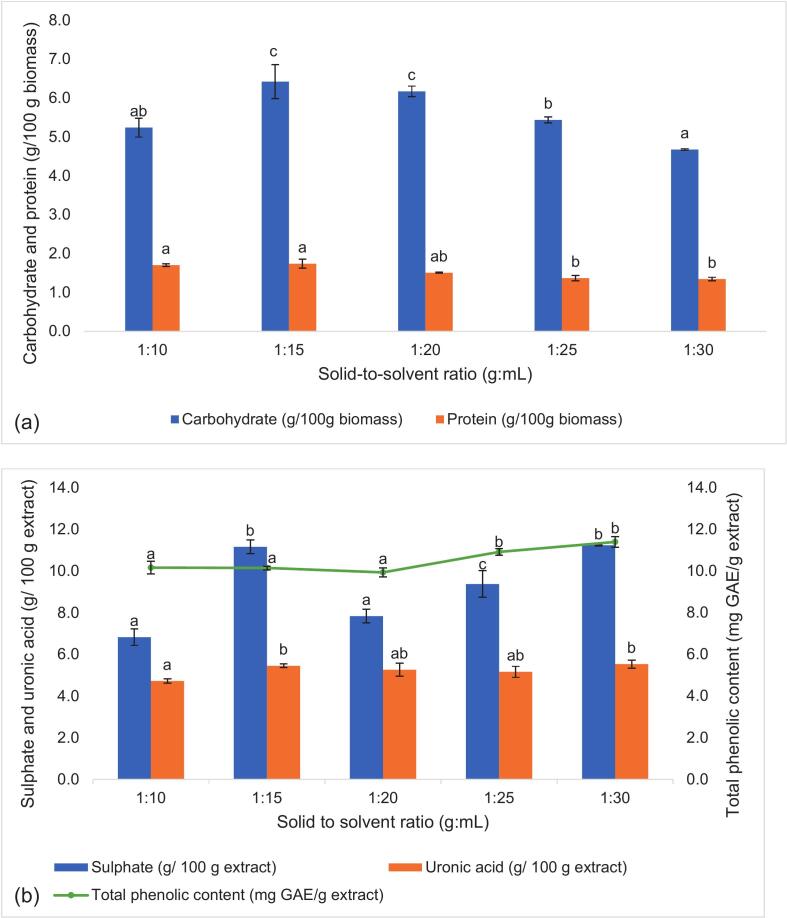
Carbohydrate and protein yield (a), along with sulphate, uronic acid and total phenolic content (b) of the microalgae extracts produced under varying solid-to-solvent ratio (1:10 to 1:30 g/mL). Data is presented as mean ± SD of three replicates. Values with different letters (^a,b,c^) within the same analysis are significantly (p < 0.05) different.

As for the solid-to-solvent (g/mL) ratio, an increase in both carbohydrate and protein yield was observed only up to a 1:15 ratio (6.426 g carbohydrate/100 g biomass), and the yield decreased as the ratio further decreased to 1:30 (Fig. 3a). Alternatively, the sulphate content generally increased as the solid-to-solvent ratio reduced from 1:20 to 1:30 (Fig. 3b). While both uronic acid and TPC did not vary significantly with the solid-to-solvent ratio, TPC showed a significant increase at 1:25 g/mL. Past studies noted that a lower solid-to-solvent ratio promoted a gradient concentration difference between the solvent and the microalgae cells, thereby increasing the mass transfer rate into the solvent [20]. However, a decrease in carbohydrate and protein yield was observed. This may be because, as the solvent volume increased, the amplitude was kept constant, thus lowering the energy per unit volume. This reduced ultrasonic energy density per unit mass of biomass, resulting in less intense cavitation and cell disruption, and subsequently lowered extraction yield [28]. A similar observation was found in past studies, where beyond a certain solid-to-solvent ratio, the extraction yield decreased, indicating that excessive solvent does not enhance extraction efficiency [11,20]. On the other hand, the hydrophilic, negatively charged exopolysaccharides (EPS) was more likely to solubilise in high volumes of water, which explains the increase in sulphate content as the solid-to-solvent ratio decreased (Fig. 3b) [29]. Additionally, the results showed that the ultrasonic conditions did not significantly affect the TPC, showing only slight improvement with more extensive extraction conditions (higher processing time, amplitude and lower solid-to-solvent ratio). The obtained TPC was lower than that reported in past studies that quantified the TPC of whole microalgae biomass. It was reported that microalgae like *N. ellipsosporum* had TPC up to 60.35 ± 2.27 mg GAE/g, whereas *C. vulgaris* had 63.5 mg GAE/g [30,31]. This could be attributed to the difference in solvent used (water) compared to past work, where less polar solvents like methanol or ethanol were typically used to maximise TPC yield during extractions [32].

### 3.2 Effect of ultrasonic processing conditions on antioxidant activity

Measuring the antioxidant activity of the extract is crucial for investigating its potential as a natural functional food ingredient for therapeutic and nutraceutical applications [1]. The antioxidant activity of microalgae extracts can be assessed using many methods, each measuring the inhibitory actions against various types of free radicals. Both DPPH and ABTS are inorganic and stable free radicals that can determine the electron-donating ability [20]. Additionally, hydroxyl (OH•) radicals are highly reactive ROS that can easily penetrate cell membranes, causing biomolecule dysfunction, which leads to tissue damage or cell death [33]. Meanwhile, the FRAP value measures the capacity to reduce ferric ions. Ferric ions are key components in ROS generation, as they react with superoxide or hydrogen peroxide molecules to form ROS.

#### 3.2.1 Processing time

The antioxidant activity of the extracts obtained under different ultrasonic processing conditions is presented in Table 1. As processing time increased, the DPPH and OH• scavenging significantly increased, reaching 91.423 % and 32.016 %, respectively, at 25 min of extraction. A similar trend in FRAP value was also observed. However, the ABTS scavenging activity was significantly lower compared to DPPH, ranging from 21.442 % (10 min) to 26.024 % (30 min). Past studies reported similar findings for sulphated polysaccharides from *C. reinhardtii* and *Dictyosphaerium* sp. polysaccharides [11,34]. The variation depended on the composition of the extracts being evaluated. Reports have found that the ABTS assay was typically highly correlated with TPC [35]. This aligns with the current findings, as the TPC of the extracts was relatively low, which in turn resulted in low ABTS inhibition. Nonetheless, based on the DPPH and ABTS assays, it can be concluded that the extract exhibited electron-donating capacities. The results also demonstrated significant effects of processing time on antioxidant activity, which contrasted with previous work reporting that extraction time did not significantly influence scavenging activity [36,37]. This may be due to the differences in the targeted compounds extracted, where smaller compounds like polyphenols require shorter extraction times compared to larger polysaccharides and proteins extracted in this study.

**Table 1:** Antioxidant activity of carbohydrate extracts produced at various ultrasonic extraction conditions.

Ultrasonic parameter	Antioxidant assay			
	DPPH scavenging (%)	OH• radical scavenging (%)	ABTS scavenging (%)	FRAP value (μmol FeSO_4_/g extract)
*Processing time (*min*)*				
10	60.237 ± 1.488^a^	19.129 ± 2.227^a^	21.442 ± 0.564^a^	46.240 ± 0.797^a^
15	90.093 ± 0.254^b^	23.401 ± 0.676^ab^	23.937 ± 0.323^ab^	49.404 ± 2.941^a^
20	86.502 ± 0.434^c^	25.373 ± 1.161^b^	22.726 ± 1.461^a^	48.085 ± 2.882^a^
25	91.423 ± 0.210^b^	32.016 ± 2.027^c^	25.822 ± 1.253^b^	58.856 ± 0.383^b^
30	86.236 ± 0.379^c^	24.115 ± 1.638^b^	26.024 ± 0.883^b^	56.355 ± 3.016^b^
*Amplitude (%)*				
20	81.782 ± 1.141^a^	16.897 ± 0.235^a^	18.630 ± 0.544^a^	46.378 ± 0.043^a^
40	81.984 ± 1.150^a^	21.584 ± 0.203^b^	24.378 ± 1.326^b^	47.201 ± 1.855^a^
60	86.502 ± 0.434^b^	25.373 ± 1.161^bc^	22.726 ± 1.461^b^	48.085 ± 2.882^a^
80	86.769 ± 0.274^b^	25.004 ± 1.022^bc^	24.708 ± 1.640^b^	50.033 ± 1.771^a^
100	89.096 ± 2.226^b^	26.600 ± 2.839^c^	22.936 ± 0.756^b^	49.100 ± 0.847^a^
*Solid-to-solvent ratio (g/mL)*				
1:10	79.187 ± 1.723^a^	23.395 ± 1.152^a^	24.881 ± 1.287^a^	50.683 ± 1.464^a^
1:15	85.971 ± 0.830^b^	34.484 ± 2.437^b^	25.113 ± 0.549^a^	51.795 ± 2.304^a^
1:20	86.502 ± 0.434^b^	25.373 ± 1.161^ac^	22.726 ± 1.461^a^	48.085 ± 2.882^ab^
1:25	87.302 ± 1.342^b^	23.225 ± 0.349^a^	24.618 ± 1.788^a^	47.739 ± 1.100^ab^
1:30	87.498 ± 0.841^b^	28.390 ± 2.788^c^	26.108 ± 2.504^a^	43.947 ± 0.910^b^

Data is presented as mean ± SD. Different alphabets (^a,b,c^) within each column for each variable indicates statistically significant difference at p < 0.05 (n = 3).

#### 3.2.2 Ultrasonic amplitude

Regarding the ultrasonic amplitude, both DPPH and OH• scavenging increased with increasing ultrasonic amplitude, reaching a maximum of 89.096 % and 26.6 %, respectively, at 100 % amplitude. ABTS activity showed no significant changes beyond 40 % amplitude, while the FRAP value was not affected by this variable. Overall, amplitude had the least effect on the antioxidant power compared to other variables. Interestingly, it was observed that increasing ultrasonic amplitude led to a higher carbohydrate, protein, sulphate and uronic acid yields, which may have contributed to the enhanced antioxidant activity. This could be due to UAE affecting the carbohydrate or protein structure, altering the functional groups exposed, which could lower antioxidant activity. Hu and Li [38] reported that while the soy protein isolate nanofibrils yield increased at 80 % amplitude, the FRAP and ABTS activities showed a significant drop due to larger cavitation bubbles altering the protein structure. Alternatively, the generation of free radicals at higher amplitudes may have degraded phenolic compounds, causing a drop in antioxidant power, which aligns with the current results as TPC decreased at 100 % amplitude [39]. Nonetheless, the significantly higher yield of functional groups attached to carbohydrates such as sulphate and uronic acid contributed to a stronger antioxidant power overall, supporting the usage of 100 % amplitude for extraction.

#### 3.2.3 Solid-to-solvent ratio

Lastly, the solid-to-solvent ratio did not significantly (p > 0.05) affect DPPH and ABTS scavenging beyond 1:15 g/mL. Conversely, OH• scavenging and FRAP value showed similar trends, where OH• scavenging peaked at 34.484 % with a 1:15 g/mL, then decreased before increasing again at 1:30 g/mL (28.390 %). The FRAP value increased to 51.795 μmol FeSO4/g extract at 1:15 g/mL, and then gradually decreased to 43.947 μmol FeSO4/g extract when the solid-to-solvent ratio was reduced to 1:30 g/mL. Again, this was consistent with the trends of the carbohydrate, protein and uronic acid yield as seen in Fig. 3a and Fig. 3b. Too high a solid-to-solvent ratio led to saturation, which lowered the yield [36]. Meanwhile, a low solid-to-solvent ratio reduced ultrasonic energy density, decreasing ultrasound power transferred to the microalgae cells [39]. With a lower cavitation effect, fewer free radicals were generated, which preserved the extract’s antioxidant activity. This explains the slight, though not significant, increase in DPPH, ABTS and OH• scavenging when the solid-to-solvent ratio lowered from 1:20 to 1:30 g/mL.

Based on the results obtained, it can be concluded that the antioxidant activity of the carbohydrates was influenced by the processing conditions, among which processing time showed the greatest changes, followed by solid-to-solvent ratio and amplitude. Hence, optimisation is essential to preserve the functional activities of the extracts. Based on both the chemical properties and antioxidant activity of the carbohydrate extracts, the optimal ultrasonic conditions were determined to be: 25 min processing time, 100 % ultrasonic amplitude and 1:15 g/mL solid-to-solvent ratio. These conditions provided the most effective antioxidant extraction, particularly for DPPH (91.423 %), OH• (34.484 %), and FRAP (58.856 μmol FeSO4/g extract) assays, indicating high radical scavenging and reducing power. Hence, these conditions were subsequently used to prepare the UAE sample, which was further analysed to compare its functionalities with the HWE extract.

### 3.3 Comparison of green technology UAE and conventional HWE extracts

#### 3.3.1 Characteristics of UAE and HWE extracts

UAE produced significantly higher yields of carbohydrate (11.615 g/100 g biomass) and protein (3.373 g/100 g biomass) compared to HWE, as shown in Table 2, corresponding to increases of 138.5 % and 178.5 %, respectively. The sulphate, uronic acid content and TPC also showed a similar trend. This demonstrated the effectiveness of ultrasonication in enhancing extraction yield from the microalgae biomass, consistent with past studies. Zhao et al. [26] reported that conventional hot water achieved a carbohydrate yield of 8.34 g/100 g biomass, whereas an optimised ultrasonic extraction obtained a maximum yield of 36.85 g/100 g biomass. The high cavitation effect effectively ruptured cell surfaces, allowing more bioactive compounds to leach into the solvent and thereby enhancing the extraction yield of sulphates and uronic acids [6]. To further characterise the carbohydrates in the extract, the fibre contents were measured. Notably, soluble fibres constituted most of the extract. The dietary fibre of UAE was slightly higher compared to HWE in terms of soluble fibres, and a significantly higher insoluble fibre was obtained for UAE. The soluble fibres could have originated from rheological polysaccharides like agar, carrageenan and alginate, which are also found in macroalgae [40]. Studies on the fibre content of microalgae remain limited, but when compared to macroalgae, a similar pattern of having higher soluble fibre (17–32 %) than insoluble fibre (5.3–16.3 %) was observed for both UAE and HWE samples [40,41]. This suggests that the extract may possess rheological or prebiotic functions due to its high soluble fibre content.

**Table 2:** Carbohydrate and protein yield, and the chemical properties of UAE and HWE extract.

Analysis	Ultrasonic extract (UAE)	Hot water extract (HWE)
Carbohydrate (g/ 100 g biomass)	11.615 ± 0.038^a^	4.870 ± 0.053^b^
Protein (g/ 100 g biomass)	3.373 ± 0.186^a^	1.211 ± 0.071^b^
Sulphate (g/ 100 g extract)	15.967 ± 0.707^a^	11.115 ± 0.006^b^
Uronic acid (g/ 100 g extract)	6.767 ± 0.152^a^	5.134 ± 0.021^b^
TPC (mg GAE/g extract)	14.778 ± 0.396^a^	13.16 ± 0.092^b^
Dietary fibre Insoluble fibre (g/ 100 g extract) Soluble fibre (g/ 100 g extract)	2.336 ± 0.683^a^ 63.583 ± 0.874^a^	0.570 ± 0.169^b^ 54.873 ± 0.300^b^

TPC = Total phenolic content.

Data is presented as mean ± SD. Different alphabets (^a,b^) within each row indicate statistically significant differences at p < 0.05 (n = 3), except for dietary fibre where n = 2.

#### 3.3.2 FTIR analysis of the extracts

**Fig. 4:**
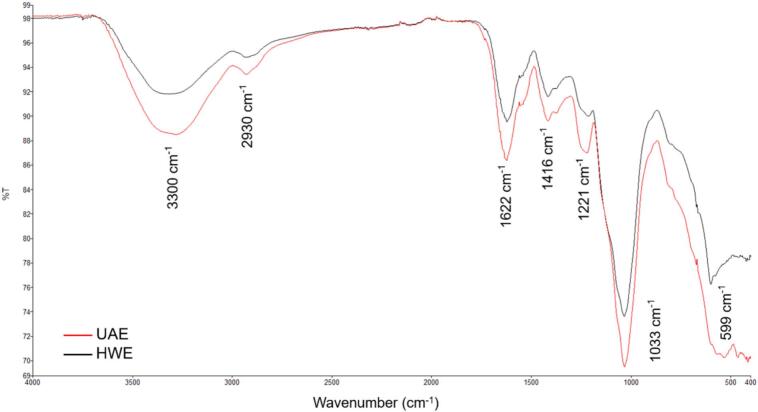
FTIR spectra of UAE (red) and HWE (black) extracts.

From the FTIR spectra of the extracts (Fig. 4), both UAE and HWE exhibited similar characteristic peaks. The absorption peaks detected at 3300 cm^−1^ and 2930 cm^−1^ correspond to O–H and C–H stretching vibration, respectively, which are typical of polysaccharide structure [3,42]. The presence of carboxylate groups (–COO), indicated by the peak at 1622 cm^−1^, accompanied by the peak at 1416 cm^−1^ corresponding to O–C=O binding, confirmed the presence of uronic acids in the extract [42,43]. Minor peaks between 1516–1526 cm^−1^ were associated with N-O stretching of nitrogenous compounds, suggesting the presence of proteins. Additionally, the band at 1221 cm^−1^ was assigned to S=O stretching vibrations, characteristic of sulphate groups [42]. Peaks around 1033 cm^−1^ correspond to C–O–C and C–O–H stretching vibrations, which are associated with glycosidic linkages within pyranose rings [5,43]. The small peaks detected at 599 and 533 cm^−1^ were associated with α and β-glycosidic, respectively [3,5]. Overall, the peaks in UAE extract were more intense than those in HWE extract, indicating a higher extraction yield, which is consistent with the results shown in Table 2.

#### 3.3.3 Antioxidant capacity of UAE and HWE extracts

**Fig. 5:**
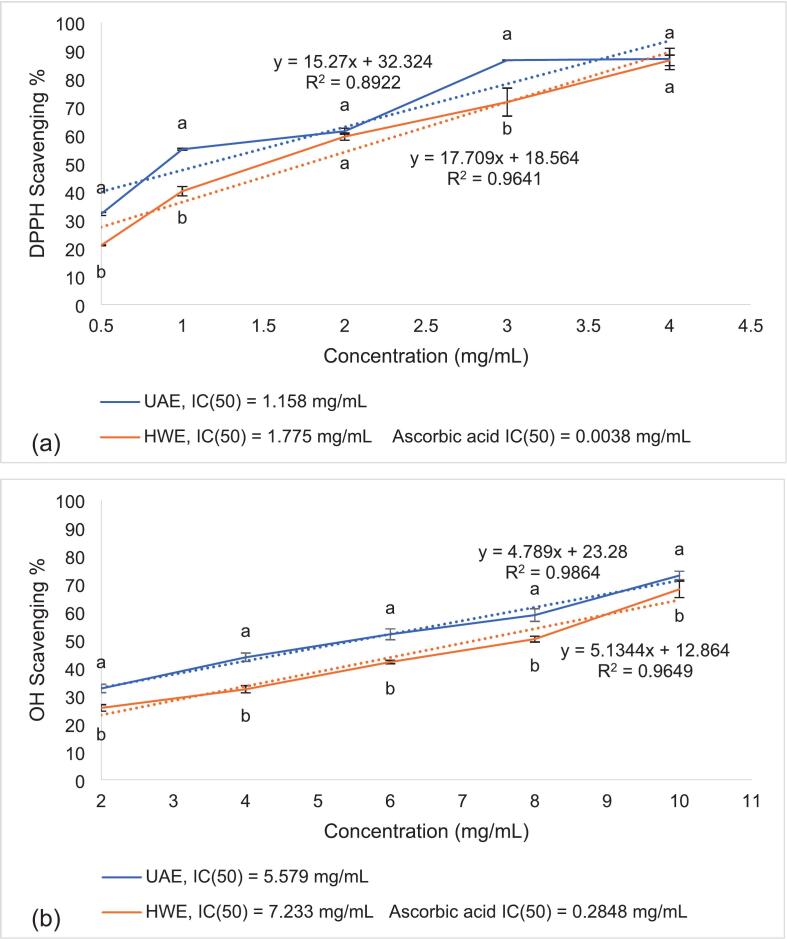

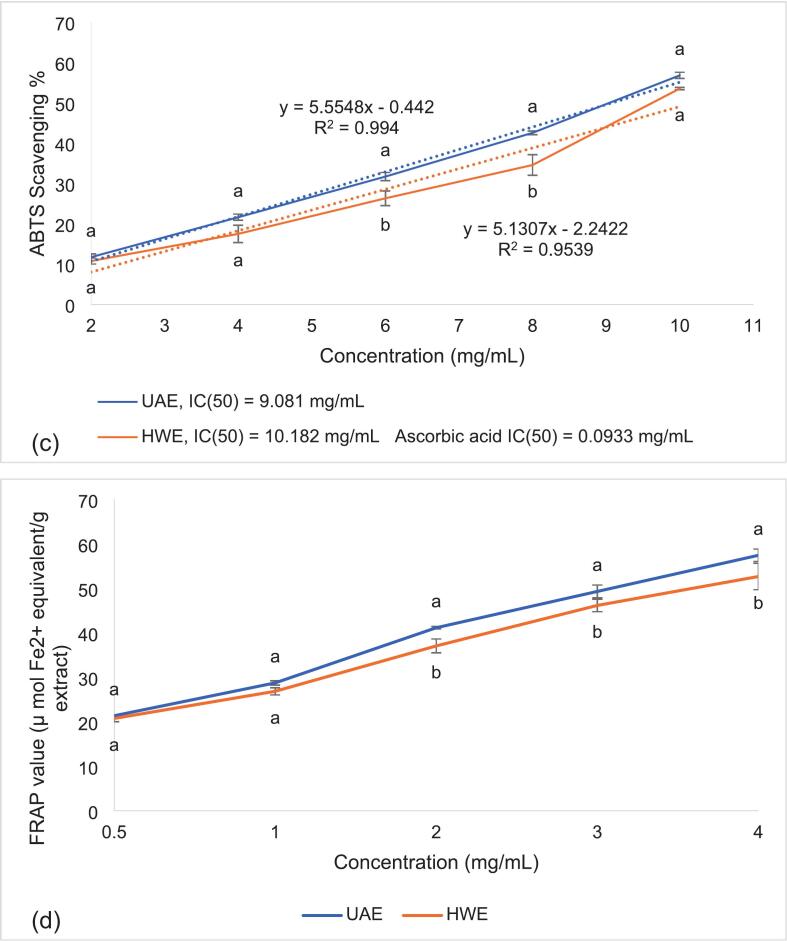
DPPH (a), OH• (b), ABTS (c) radical scavenging activity, and FRAP value (d) of UAE and HWE extracts at varying concentrations (0.5 to 10 mg/mL), along with IC_50_ of both extracts and ascorbic acid listed in the legends. Data are mean ± SD of three replicates. Data points between the two different samples at the same concentration with different letters (^a,b^) are significantly (p < 0.05) different.

The antioxidant activities of the UAE and HWE extracts were investigated at increasing concentrations to identify and compare their half-maximal inhibitory concentration (IC_50_), which represents the concentration needed to inhibit radical activity by 50 %. The antioxidant activity of UAE was generally higher than HWE (except for DPPH activity) (Fig. 5), demonstrating the advantages of ultrasonication over conventional extraction methods.

For DPPH scavenging, UAE showed no significant difference compared to HWE at 4 mg/mL (Fig. 5a), suggesting that the electron-donating capacity of both extracts peaked around 4 mg/mL. However, the IC_50_ of UAE (1.158 mg/mL) was lower compared to HWE (1.775 mg/mL), indicating that UAE exhibited higher antioxidant activity at lower concentrations. For comparison, the IC_50_ of ascorbic acid was 0.038 mg/mL, confirming that while the extracts were less potent than the pure standard, they still demonstrated notable radical scavenging ability. Nonetheless, at this concentration (4 mg/mL), the extract also showed improved DPPH scavenging compared to past studies. For instance, polysaccharides purified from Arctic *Chlorella* sp. showed 60.20 % inhibition at 5 mg/mL, while *N. commune* polysaccharides showed a 57 % scavenging capacity at 4 mg/mL [20,33].

Additionally, UAE showed significantly stronger OH• scavenging than HWE (Fig. 5b), although a higher concentration was needed compared to DPPH scavenging (IC_50_ = 5.579 mg/mL). For reference, the IC_50_ of ascorbic acid for OH• radical was 0.2848 mg/mL, indicating that the extract’s activity was moderate but still comparable to polysaccharides reported in previous studies, which showed approximately 40 % activity at 4 mg/mL [33]. Chen et al. [11] extracted polysaccharides from *Dictyosphaerium* sp. and found a scavenging activity of 86.35 % at 10 mg/mL. Similarly, EPS from *Botryococcus braunii* demonstrated 70 % OH• scavenging at 5 mg/mL [44].

In terms of ABTS scavenging, UAE achieved 56.808 % inhibition at 10 mg/mL (Fig. 5c). Although this activity was lower than DPPH scavenging, the results obtained are still higher compared to previous studies. Polysaccharide samples generally show stronger DPPH than ABTS activity, where an ABTS scavenging activity of 25–27 % at 10 mg/mL was recorded [11,44]. In contrast, UAE achieved comparable ABTS activity at lower concentrations. Another study, however, reported an activity of 81 % at 6 mg/mL, which could be due to differences in microalgae species, where the extract produced from *C. reinhardtii* had a sulphate content of 33 %, contributing to a strong electron-donating capacity [34].

Similarly, the FRAP value of the UAE sample (57.474 μmol FeSO4/g) was slightly higher than that of HWE (52.707 μmol FeSO4/g) at 4 mg/mL (Fig. 5d). Extracts from other species have shown higher FRAP values (>100 μmol FeSO4/g). For instance, *Chlorella* sp. extracts generally display low FRAP activity due to having lower TPC [45]. This suggests the extract has relatively weak reducing power in acidic conditions [11,45].

Overall, UAE produced a crude carbohydrate extract with higher antioxidant activity compared to conventional HWE. Its enhanced extraction efficiency allowed more bioactive compounds to be released, contributing to greater antioxidant performance. Similar findings have been reported previously, where ultrasonication increased the yield of carbohydrates containing functional groups like sulphate, uronic acid, and phenolic compounds [5,6]. Notably, this study demonstrates that ultrasonication conducted at ambient temperatures can effectively preserve the bioactivities of the extract.

### 3.4 SEM of microalgae biomass before and after extraction

**Fig. 6:**
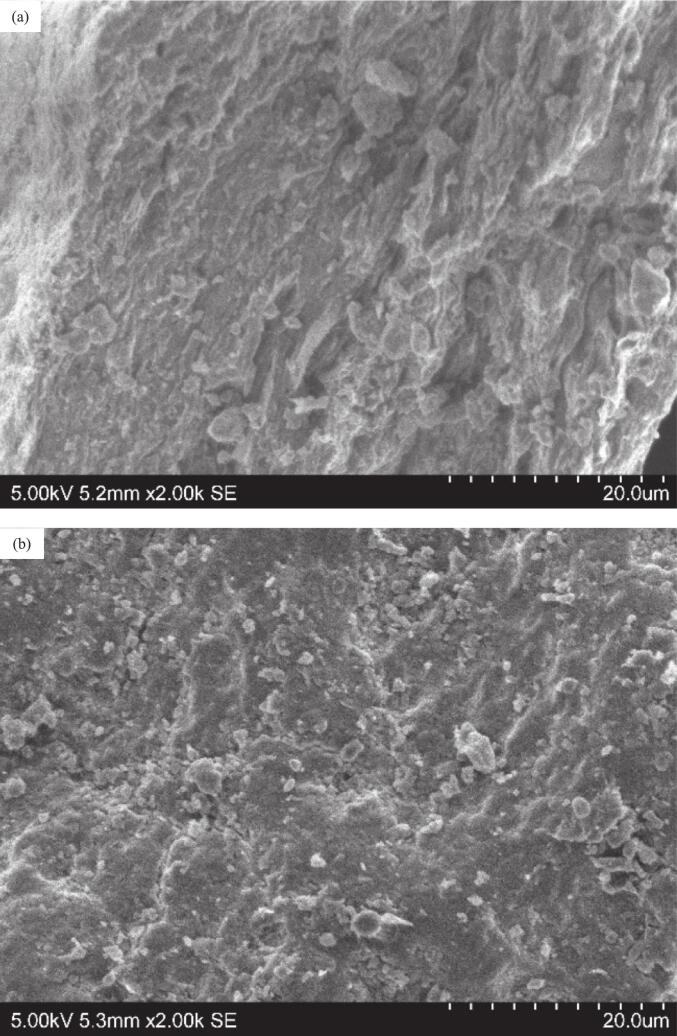

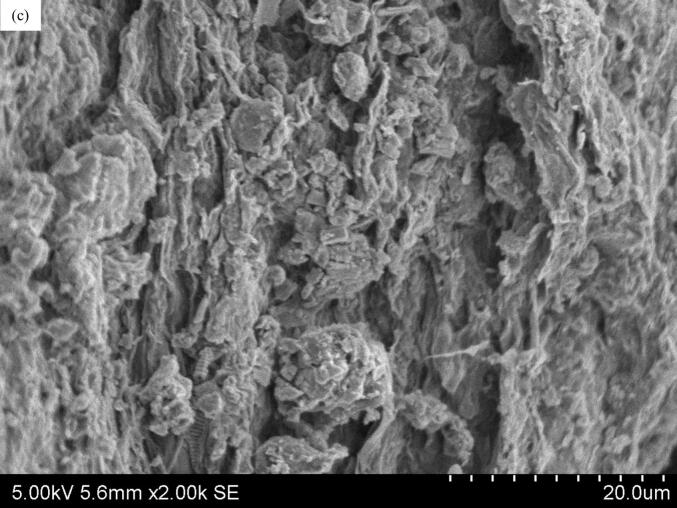
SEM images of microalgae biomass powder before (a) and after HWE (b) and UAE (c) treatment at ×2000 magnification.

The surface morphologies of the microalgae biomass before and after UAE and HWE are shown in Fig. 6. After HWE (Fig. 6b), cracks and microscopic fragmentation appeared on the cell surface, due to the heat-induced softening of the cell walls, allowing the solvent to penetrate the cells. In contrast, UAE produced a rougher and more fragmented cell surface (Fig. 6c), demonstrating the effectiveness of UAE in cell disruption. The rapid collapse of microbubbles generates high localised pressure, damaging the cell surface at multiple sites simultaneously. This resulted in a coarse surface that increased the surface area, facilitating solvent permeation and enhancing biomolecule extraction [10]. This explains the higher extraction yield obtained in this study, while the shorter extraction time and ambient temperature of UAE helped preserve extract bioactivities, leading to higher antioxidant activity.

### 3.5 Correlation between physical and chemical properties and antioxidant activity

The Pearson correlation coefficients between the physicochemical properties and antioxidant activities of the extracts were determined to identify the factors contributing to their functionality (Table 3). The carbohydrate yield showed a significant positive correlation with the protein, sulphate and uronic acid contents, indicating that these functional groups were co-extracted along with the carbohydrates, as they are commonly found in microalgae EPS [46]. However, the sulphate content exhibited only a weak correlation with antioxidant activity; only DPPH and OH• scavenging were significant, but with low correlation values (r < 0.5). In contrast, the protein and uronic acid contents were strongly correlated (r > 0.6) with the antioxidant activity (except for FRAP). The carboxyl groups of uronic acids enhance electrostatic attraction, allowing the functional group to act as electron‑donating sites that can neutralise free radicals [47]. Meanwhile, microalgae proteins contain phycobilin groups with conjugated double bonds and exposed aromatic residues that can donate electrons or hydrogen atoms to quench ROS [48]. Furthermore, TPC also showed a positive correlation with all antioxidant assays. This is consistent with past studies demonstrating a strong positive relationship between TPC and ABTS and FRAP activities [35,49]. Based on the Pearson correlation results, the antioxidant activity of the extract was mainly driven by uronic acid content > TPC > protein content > sulphate content.

**Table 3:** Pearson correlation coefficients between various chemical properties of the carbohydrate extract and antioxidant activities.

** Correlation is significant at the 0.01 level (2-tailed).

* Correlation is significant at the 0.05 level (2-tailed).

Carb = Carbohydrate content, Protein = Protein content, Sul = Sulphate content, Uro = Uronic acid content, TPC = Total phenolic content, DPPH = DPPH scavenging, ABTS = ABTS scavenging, OH = OH• radical scavenging, FRAP = FRAP value.

## 4 Conclusion

This study demonstrated that ultrasonic-assisted extraction (UAE) under optimal conditions (25 min, 100 % amplitude, 1:15 g/mL) significantly improved carbohydrate recovery and antioxidant activity compared with hot water extraction, yielding approximately 138 % more carbohydrates and about 35 % stronger DPPH scavenging activity (p < 0.05). Among the extraction variables, processing time had the strongest influence on antioxidant activity, followed by the solid-to-solvent ratio and amplitude. Uronic acid was identified as the main contributor to antioxidant activity, highlighting the value of UAE in enhancing both yield and functionality of microalgae-derived carbohydrates. Beyond these findings, UAE presents potential for industrial application as a green extraction strategy for microalgae carbohydrates. Future studies should focus on bioactivity-focused purification of the carbohydrate fractions and explore additional bioactivities beyond antioxidant potential. Additionally, upscaling and integration of UAE with other sustainable processing methods, such as pulsed electric fields, should be investigated to further support the development of microalgae carbohydrates as functional food ingredients.

## Funding source

The research study was funded by High Impact Research Support Fund 2024 and School of Science Graduate Research Funding, Monash University Malaysia.

## CRediT authorship contribution statement

**Eldwin Ze Hao Ooi:** Writing – original draft, Methodology, Investigation, Formal analysis, Data curation, Conceptualization. **Eng-Seng Chan:** Writing – review & editing, Validation, Supervision, Resources, Project administration, Conceptualization. **Cher Pin Song:** Writing – review & editing, Validation, Supervision, Resources, Project administration, Conceptualization. **Janarthanan Pushpamalar:** Writing – review & editing, Validation, Supervision. **Yee-Ying Lee:** Writing – review & editing, Validation, Supervision, Resources, Project administration, Conceptualization.

## Declaration of competing interest

The authors declare that they have no known competing financial interests or personal relationships that could have appeared to influence the work reported in this paper.

## References

[1] Gao L., Qin Y., Zhou X., Jin W., He Z., Li X., Wang Q. (2024). Microalgae as future food: rich nutrients, safety, production costs and environmental effects. Sci. Total Environ..

[2] de Carvalho Silvello M.A., Severo Gonçalves I., Patrícia Held Azambuja S., Silva Costa S., Garcia Pereira Silva P., Oliveira Santos L., Goldbeck R. (2022). Microalgae-based carbohydrates: a green innovative source of bioenergy. Bioresour. Technol..

[3] Liu F., Chen H., Qin L., Al-Haimi A.A.N.M., Xu J., Zhou W., Zhu S., Wang Z. (2023). Effect and characterization of polysaccharides extracted from *Chlorella* sp. by hot-water and alkali extraction methods. Algal Res..

[4] Severo I.A., Dias R.R., do Nascimento T.C., Deprá M.C., Maroneze M.M., Zepka L.Q., Jacob-Lopes E. (2022). Microalgae-derived polysaccharides: potential building blocks for biomedical applications. World J. Microbiol. Biotechnol..

[5] Wang X., Yang Z., Liu Y., Wang X., Zhang H., Shang R., Laba C., Wujin C., Hao B., Wang S. (2022). Structural characteristic of polysaccharide isolated from *Nostoc commune*, and their potential as radical scavenging and antidiabetic activities. Sci. Rep..

[6] Yu M., Chen M., Gui J., Huang S., Liu Y., Shentu H., He J., Fang Z., Wang W., Zhang Y. (2019). Preparation of *Chlorella vulgaris* polysaccharides and their antioxidant activity in vitro and in vivo. Int. J. Biol. Macromol..

[7] Nagahawatta D.P., Liyanage N.M., Jayawardena T.U., Yang F., Jayawardena H.H.A.C.K., Kurera M.J.M.S., Wang F., Fu X., Jeon Y.-J. (2023). Functions and values of sulfated polysaccharides from seaweed. Algae.

[8] Moreira J.B., Vaz B.D.S., Cardias B.B., Cruz C.G., Almeida A.C.A., Costa J.A.V., Morais M.G. (2022). Microalgae polysaccharides: an alternative source for food production and sustainable agriculture. Polysaccharides.

[9] Gouda M., Tadda M.A., Zhao Y., Farmanullah F., Chu B., Li X., He Y. (2022). Microalgae bioactive carbohydrates as a novel sustainable and eco-friendly source of prebiotics: emerging health functionality and recent technologies for extraction and detection. Front. Nutr..

[10] Liu Y., Liu X., Cui Y., Yuan W. (2022). Ultrasound for microalgal cell disruption and product extraction: a review. Ultrason. Sonochem..

[11] Chen C., Zhao Z., Ma S., Rasool M.A., Wang L., Zhang J. (2020). Optimization of ultrasonic-assisted extraction, refinement and characterization of water-soluble polysaccharide from *Dictyosphaerium* sp. and evaluation of antioxidant activity in vitro. J. Food Meas. Charact..

[12] Olia M.S.J., Azin M., Moazami N. (2023). Comparison of different pretreatment methods to facilitate the carbohydrate release from two microalgae isolates: a critical step in bioethanol production. Biomass Convers. Biorefin..

[13] Hosni S., Gani S.S.A., Orsat V., Hassan M., Abdullah S. (2023). Ultrasound-assisted extraction of antioxidants from *Melastoma malabathricum* Linn.: Modeling and optimization using Box–Behnken design. Molecules.

[14] Aloisio C., Razola-Díaz M.D.C., Aznar-Ramos M.J., Longhi M.R., Andreatta A.E., Verardo V. (2023). Optimization of the extraction conditions of bioactive compounds from *Ocimum basilicum* leaves using ultrasound-assisted extraction via a sonotrode. Molecules.

[15] DuBois M., Gilles K.A., Hamilton J.K., Rebers P.A., Smith F. (1956). Colorimetric method for determination of sugars and related substances. Anal. Chem..

[16] Lourenço S.O., Barbarino E., Lavín P.L., Lanfer Marquez U.M., Aidar E. (2004). Distribution of intracellular nitrogen in marine microalgae: calculation of new nitrogen-to-protein conversion factors. Eur. J. Phycol..

[17] Torres P., Nagai A., Palacios Jara C.E., Santos J., Chow F., Santos D. (2021). Determination of sulfate in algal polysaccharide samples: a step-by-step protocol using microplate reader. Ocean Coast. Res..

[18] Blumenkrantz N., Asboe-Hansen G. (1973). New method for quantitative determination of uronic acids. Anal. Biochem..

[19] Khawli F.A., Martí-Quijal F.J., Pallarés N., Barba F.J., Ferrer E. (2021). Ultrasound extraction mediated recovery of nutrients and antioxidant bioactive compounds from *Phaeodactylum tricornutum* microalgae. Appl. Sci..

[20] Song H., He M., Gu C., Wei D., Liang Y., Yan J., Wang C. (2018). Extraction optimization, purification, antioxidant activity, and preliminary structural characterization of crude polysaccharide from an Arctic *Chlorella* sp. Polym.

[21] Lupatini A.L., de Oliveira Bispo L., Colla L.M., Costa J.A.V., Canan C., Colla E. (2017). Protein and carbohydrate extraction from *S. platensis* biomass by ultrasound and mechanical agitation. Food Res. Int..

[22] Vignaud J., Loiseau C., Hérault J., Mayer C., Côme M., Martin I., Ulmann L. (2023). Microalgae produce antioxidant molecules with potential preventive effects on mitochondrial functions and skeletal muscular oxidative stress. Antioxidants.

[23] Wang P., Cheng C., Ma Y., Jia M. (2020). Degradation behavior of polyphenols in model aqueous extraction system based on mechanical and sonochemical effects induced by ultrasound. Sep. Purif. Technol..

[24] Aktı N., Yildiz S. (2025). Exploring ultrasound-induced free radical formation: a comparative study in water and sour cherry juice using glutathione and terephthalic acid indicators. Ultrason. Sonochem..

[25] Wang F., Ma Y., Liu Y., Cui Z., Ying X., Zhang F., Linhardt R.J. (2017). A simple strategy for the separation and purification of water-soluble polysaccharides from the fresh *Spirulina platensis*. Sep. Sci. Technol..

[26] Zhao G., Chen X., Wang L., Zhou S., Feng H., Chen W.N., Lau R. (2013). Ultrasound assisted extraction of carbohydrates from microalgae as feedstock for yeast fermentation. Bioresour. Technol..

[27] Ji D., Wang Q., Lu T., Ma H., Chen X. (2022). The effects of ultrasonication on the phytochemicals, antioxidant, and polyphenol oxidase and peroxidase activities in coffee leaves. Food Chem..

[28] Baltrusch K.L., Torres M.D., Domínguez H. (2025). Optimizing ultrasound-assisted extraction with custom design and response surface methodology: a case study using *Ulva* spp. Ultrason. Sonochem..

[29] Raposo M.F., de Morais R.M., Bernardo de Morais A.M. (2013). Bioactivity and applications of sulphated polysaccharides from marine microalgae. Mar. Drugs.

[30] Dadwar A., Sibi G. (2019). In vitro antioxidant and anti-diabetic potential of green microalgae, *Chlorella vulgaris*. Adv. Biol. Res..

[31] Li H.-B., Cheng K.-W., Wong C.-C., Fan K.-W., Chen F., Jiang Y. (2007). Evaluation of antioxidant capacity and total phenolic content of different fractions of selected microalgae. Food Chem..

[32] Monteiro M., Santos R.A., Iglesias P., Couto A., Serra C.R., Gouvinhas I., Barros A., Oliva-Teles A., Enes P., Díaz-Rosales P. (2020). Effect of extraction method and solvent system on the phenolic content and antioxidant activity of selected macro- and microalgae extracts. J. Appl. Phycol..

[33] Quan Y., Yang S., Wan J., Su T., Zhang J., Wang Z. (2015). Optimization for the extraction of polysaccharides from *Nostoc commune* and its antioxidant and antibacterial activities. J. Taiwan Inst. Chem. Eng..

[34] Kamble P., Cheriyamundath S., Lopus M., Sirisha V.L. (2018). Chemical characteristics, antioxidant and anticancer potential of sulfated polysaccharides from *Chlamydomonas reinhardtii*. J. Appl. Phycol..

[35] Floegel A., Kim D.-O., Chung S.-J., Koo S.I., Chun O.K. (2011). Comparison of ABTS/DPPH assays to measure antioxidant capacity in popular antioxidant-rich US foods. J. Food Compos. Anal..

[36] Aliaño-González M.J., Barea-Sepúlveda M., Espada-Bellido E., Ferreiro-González M., López-Castillo J.G., Palma M., Barbero G.F., Carrera C. (2022). Ultrasound-assisted extraction of total phenolic compounds and antioxidant activity in mushrooms. Agronomy.

[37] Chen X., Qi Y., Zhu C., Wang Q. (2019). Effect of ultrasound on the properties and antioxidant activity of hawthorn pectin. Int. J. Biol. Macromol..

[38] Hu A., Li L. (2022). Effects of ultrasound pretreatment on functional property, antioxidant activity, and digestibility of soy protein isolate nanofibrils. Ultrason. Sonochem..

[39] Dzah C.S., Duan Y., Zhang H., Wen C., Zhang J., Chen G., Ma H. (2020). The effects of ultrasound assisted extraction on yield, antioxidant, anticancer and antimicrobial activity of polyphenol extracts: a review. Food Biosci..

[40] Lahaye M. (1991). Marine algae as sources of fibres: determination of soluble and insoluble dietary fibre contents in some ‘sea vegetables’. J. Sci. Food Agric..

[41] Nova P., Pimenta A., Maricato É., Nunes C., Abreu H., Coimbra M., Freitas A., Gomes A. (2023). Chemical composition and antioxidant potential of five algae cultivated in fully controlled closed systems. Molecules.

[42] Elain A., Nkounkou C., Le Fellic M., Donnart K. (2020). Green extraction of polysaccharides from *Arthrospira platensis* using high pressure homogenization. J. Appl. Phycol..

[43] Mousavian Z., Safavi M., Azizmohseni F., Hadizadeh M., Mirdamadi S. (2022). Characterization, antioxidant and anticoagulant properties of exopolysaccharide from marine microalgae. AMB Expr..

[44] Wang W.-N., Li T., Li Y., Zhang Y., Wu H.-L., Xiang W.-Z., Li A.-F. (2022). Exopolysaccharides from the energy microalga strain *Botryococcus braunii*: purification, characterization, and antioxidant activity. Foods.

[45] Tiong I.K.R., Nagappan T., Abdul Wahid M.E., Tengku Muhammad T.S., Tatsuki T., Satyantini W.H., Mahasri G., Sorgeloos P., Sung Y.Y. (2020). Antioxidant capacity of five microalgae species and their effect on heat shock protein 70 expression in the brine shrimp *Artemia*. Aquac. Rep..

[46] Magnabosco C., Santaniello G., Romano G. (2025). Microalgae: a promising source of bioactive polysaccharides for biotechnological applications. Molecules.

[47] Hu Y.-B., Hong H.-L., Liu L.-Y., Zhou J.-N., Wang Y., Li Y.-M., Zhai L.-Y., Shi Z.-H., Zhao J., Liu D. (2022). Analysis of structure and antioxidant activity of polysaccharides from *Aralia continentalis*. Pharmaceuticals.

[48] Coulombier N., Jauffrais T., Lebouvier N. (2021). Antioxidant compounds from microalgae: a review. Mar. Drugs.

[49] Ma Y., Huang H. (2014). Characterisation and comparison of phenols, flavonoids and isoflavones of soymilk and their correlations with antioxidant activity. Int. J. Food Sci. Tech..

