# Supplementary material for: Ultrasound-assisted extraction enhances recovery of antioxidant-rich carbohydrate fraction from mixed microalgae species
Source: Ultrason Sonochem. 2025 Oct 30;123:107656. doi: 10.1016/j.ultsonch.2025.107656 (PMC12637094; doi:10.1016/j.ultsonch.2025.107656)
Supplement: Supplementary Data 1 [file mmc1.docx]

Figure S1. Calibration curves for the determination of (a) sulphate content (sodium sulphate standard), (b) uronic acid content (glucuronic acid standard), (c) total phenolic content (gallic acid standard), and (d) ferric reducing antioxidant power FRAP value (iron (II) sulphate standard). Each calibration curve was constructed based on mean absorbance (n = 3), with linear regression equations and R² values shown in the respective panels.
